# ANPEP: A Potential Regulator of Tumor Cell—Immune Metabolic Interactions in Prostate Cancer of Men of African Descent

**DOI:** 10.1200/GO.22.58000

**Published:** 2022-05-05

**Authors:** Asmaa Elkenawi, Shivanshu Awasthi, Amparo Serna, Jasreman Dhillon, Kosj Yamoah

**Affiliations:** ^1^ Moffitt Cancer Center, Tampa, FL

## Abstract

Prostate cancer (PCa) is the leading cause of male cancer deaths in Africa. In USA, African American men (AA) experience higher mortality rate compared to European American men (EA). While socioeconomics factors contribute toward these disparities, distinct molecular mechanisms in AA remain important drivers. For example, in previous work, we discovered that PCa form AA men harbored a significantly higher expression of Aminopeptidase N (ANPEP) as compared with EA. Aminopeptidase N regulates immune cell function and mediates tumor cell migration but its role in PCa remains uninvestigated.

**METHODS:**

We explored ANPEP expression related to a range of genomic classifiers in multiple datasets. We then prospectively examined the differentially expressed genes in PCa tissues collected from 120 AA and 120 EA patients enrolled on the VANDAAM clinical trial. We further correlated ANPEP expression with various signatures related to immune and metabolic activity. We also experimentally examined the expression of ANPEP in different components of tumor microenvironment.

**RESULTS:**

In the VANDAAM study, ANPEP represents the top differentially expressed gene between AA and EA men. Our data driven approach revealed that ANPEP correlates with signature of cholesterol transport and androgen signaling. Interestingly, these signatures dominate in PCa with high macrophage infiltration. Deconvolution of immune cell content using computational approaches indeed demonstrated that only AA patients with high ANPEP expression significantly accumulated high content of inflammatory macrophages. Further experimental findings showed that ANPEP indeed represents a marker of tumor-associated macrophages, a type of immune cells known to contribute to PCa progression.

**CONCLUSION:**

We have identified ANPEP as a macrophage-related gene with a potential role in cholesterol metabolism and AR signaling in AA. Future work will focus on the functional role of ANPEP activity in the tumor immune microenvironment using Liquid chromatography-high resolution mass spectrometry and explants derived from AA and EA PCa patients.

